# Financial Integration Scenario for Community Integrated Care: Focusing on the Case of Korea

**DOI:** 10.5334/ijic.6465

**Published:** 2022-05-31

**Authors:** Byunggeor Moon

**Affiliations:** 1 Sungkyunkwan University, School of Global Leader & Department of Public Administration & Graduate School of Governance, South Korea

**Keywords:** community care, financial integration, public finance, health insurance, long-term care

## Abstract

This study analyzes the potential effects of a reform based on community integrated care in Korea, which is experiencing a high rate of population aging, and a specific method for financial integration. We first analyze the size and trend of funds used for the care for the elderly out of Korea’s health insurance, long-term care insurance, and national budget. We then analyze the amount of financial resources required and the cost-saving effect when the related financial resources are converted into local community care funds. This approach sheds light on the possibility of harmonizing healthcare policy for the elderly and integrated care under the existing insurance system and suggests a direction for reform in policies pertaining to healthcare for the elderly. Given that the same services are provided, we find that combining the finances from the insurance and the national budget would result in a fund of KRW 2.6 trillion to KRW 4.7 trillion. This approach also confirms that health care costs for the elderly can be reduced by 1-5% in the long term, which we estimate to be between KRW 1 trillion to KRW 4 trillion by 2050. We find that by tapping into the national budget to manage the pre-medical stage care, we can utilize an efficient operation mechanism unlike insurance. We also confirm that information exchange and harmonious operation between the national budget and state-run insurance as well as feedback and incentives through performance management are necessary for these results to become a reality.

## 1 Introduction

Korea is experiencing a high rate of population aging and a low fertility rate at the same time. According to the Organization for Economic Co-operation and Development (OECD) Demography statistics,^1^ from 1970 to 2018, the average annual increase in Korea’s aging rate was 3.3%, the fastest among 37 OECD countries. In particular, Korea entered the category of an “aging society” (defined as having more than 7% of the population as the elderly) in 2000 and entered an “aged society” (having more than 14% of the population as the elderly) in 2018, 18 years later. If this trend continues, the OECD predicts that Korea will enter a “super-aged society” (having more than 20% of the population as the elderly) in 2026, eight years after entering the aged society. Comparing this trend with Japan, Italy, and Spain, which have the highest proportion of the elderly population among OECD member countries, Korea is expected to overtake Italy, the third highest in the OECD, in 2036 with the fastest rise in the aging rate. The OECD predicts that by 2050, the country will become the second most-aged country in the OECD with an elderly population of 37.4%, which is only 0.3% lower than Spain’s 37.7%.

Furthermore, according to OECD 2018 statistics, Korea’s total fertility rate decreased by an average of 3.1% annually from 4.53 in 1970 to 0.98 in 2018, indicating that the decrease in fertility rate is the fastest among the 37 OECD countries. The yearly trend shows that, in 1984, the number was 1.74, lower than the US (1.81); in 1993, 1.65, lower than France (1.66); and in 2001, 1.31, lower than Japan (1.33). Accordingly, the total dependency ratio in Korea, which is defined as the population that 100 working-age people must support, was 38.6 in 2020 but will exceed 77.5 in 2040 and will rapidly increase to 108.2 in 2060. The youth dependency ratio is expected to remain unchanged at 16.9 in 2020 and 16.7 in 2060. The old-age dependency ratio is expected to increase 4.2 times compared to 2020, from 21.7 in 2020 to 60.1 in 2040 and 91.4 in 2060. A decrease in the total population, mainly due to the decrease in the working-age population, and a sharp rise in the population support ratio due to an increase in the elderly population are expected to accelerate the deterioration of Korea’s social welfare-related finances. In particular, in the process of experiencing such rapid aging and low fertility at the same time, the national expenditure on health and social care for the elderly is expected to increase rapidly [1,2,3].

To deal with the expected increase in healthcare expenditure for the elderly, Korea provides financial support for the care and support of the elderly through the dual system of the National Health Insurance and Long-term Care Insurance. The National Health Insurance has been in operation since 1989 to cover the entire nation. However, given the situation in Korea, which is experiencing a combination of a rapidly aging population and a low birth rate, the Korean government introduced a long-term care insurance for the elderly in 2008 [4]. However, medical expenses for people over the age of 65 are increasing rapidly. As of 2019, out of KRW 31.9 trillion in medical expenses allocated for the National Health Insurance, KRW 16.2 trillion was used for medical expenses for those over 65 years old, accounting for about 50.8%. Furthermore, between 2014 and 2018, long-term care expenses for the elderly increased by 16.1% each year on average. As a result, we expect that funding for the long-term care insurance will be depleted by 2030 based on the current premium rate and the current trend [5].

In order to provide effective health care while responding to rapidly increasing medical expenses, many governments worldwide are implementing various policies. As one of the major trends, policies that integrate medical and social care are being implemented. The reason for these policies and reforms is to effectively manage limited financial resources and ongoing financial expenditures related to elderly health and to provide effective services through the integration of segmented services [6,7]. Different approaches using various integration models are being tested for the integration of health and social care [8]. For example, in the United Kingdom, where there is a medical service system similar to that of Korea, efforts are being made to pursue cost-effectiveness through financial integration of health and social care. In particular, the Better Care Fund, which combines medical insurance and social care fund, has been established. Empirical analysis shows that there this policy led to a reduction of hospitalization rate and costs [9].

This study analyzes the characteristics of Korea’s National health insurance and long-term care insurance in terms of their overlap with social care in caring for the elderly. The purpose of this study is to examine the potential financial outcomes and the sustainability of implementing the British policy of integrating healthcare and social care in Korea. Furthermore, we predict the size of the integrated funds through this approach and analyze the operational and policy design requirements for successful integration through a case study of an existing financially integrated social care policy. Many countries, including Korea, are spending a lot of money on healthcare and social care due to the rapidly aging population, and the scale is expected to increase in the future. In such a situation, it is important to suggest a direction for reform through institutional analysis and to make a financial estimate based on the analysis of existing cases. Based on this, we find it meaningful to present an alternative policy that can be used for reform in the medical welfare field and to provide a policy direction for other countries that have become an aging society. The paper is organized as follows. Section 2 discusses an overview of the structure and the budget size of welfare programs, national health insurance, and long-term care insurance for the elderly in Korea. Section 3 discusses the parts of the health insurance and long-term care insurance businesses that can be transferred to social care and examines the scale of financial integration and cost reduction. Section 4 draws the policy implications of these predictions and the prerequisites for reducing costs while having an effective fiscal integration for integrated care.

## 2 Overview of the Health and Social Care for Elderly in Korea

### 2.1 National Health Insurance

The Medical Insurance Act in Korea was enacted in 1963, and Korea introduced compulsory social health insurance for industrial workers in large enterprises with 500 or more employees in 1977. After that, the coverage of insurance was gradually expanded to include the self-employed, and by 1989, the entire population was covered. Thus, only 12 years after the introduction of social health insurance, Korea achieved universal coverage of its population. In Korea’s national health insurance, insurance claims review and payment to medical personnel were centralized. Although the statutory pay packages were the same across schemes, there were several insurance unions that individually covered employees and the self-employed. In 2000, there was a major change in the structure of the health insurance program, and all insurance associations were consolidated into one [10].

Due to the rapidly aging population, medical expenses for the elderly population are increasing rapidly. The health insurance payouts for inpatient treatments in all hospitals totaled about KRW 31.92 trillion in 2019 – an annual increase of 18.0% from KRW 11.17 trillion in 2008 – of which KRW 16.23 trillion, accounting for about 50.8% was for those over 65 years of age.

Long-term care Hospitals (LTCH) offers another form of LTC in Korea. They offer a variety of health care services, including subacute Long-term care (LTC), palliative care, and rehabilitation services. As of June 2020, there were 1,481 LTC Hospitals in Korea. LTCHs are covered by the NHI and focuses on medical and functional rehabilitation to help patients return home. Although LTCHs and nursing homes have different operating entities, resources, and objectives, some studies have found little difference in function between LTCHs and nursing homes. Additionally, the Health Insurance Review & Assessment Service (HIRA) introduced a health insurance fee system for LTCHs in 2008 by requiring LTCHs to classify inpatients. Along with the aging population, the number of nursing hospitals increased by 11% to 438,563 between 2008 and 2017, and the number of beds increased by 17.4% to 289,227 between 2008 and 2017. In addition, the inpatient treatment cost of nursing hospitals for health insurance benefits was KRW 5.75 trillion in 2019, an increase of 209% from 0.96 trillion KRW in 2008, showing a rapid increase.

In particular, in the case of LTC hospitals, patients are divided into groups based on their condition and medical need. Before 2019, patients were divided into 7 groups. Since then, however, patients began to be separated into 5 groups based on their medical needs. Table 1 shows the five patient groups and the number of patients in each group. The category of social admission (elective hospitalization) groups patients that neither fall under the highest nor mild medical condition and believe that they need some hospitalization but have a low medical need. For these patients, the co-payment rate is 40%, and they may be hospitalized for a certain period of time.

**Table 1:** Estimate of Ratio of Patient group in new scheme.


PATIENT GROUP	NUMBER OF PATIENT	RATIO (%)

Total	438,563	100.0

Top-level medical needs	8,022	1.8

High-level medical needs	100,544	22.9

Middle-level medical needs	108,697	24.8

Light-level medical needs	47,596~99,848	11.2~22.8

Social Admission	121,452~173,704	27.7~39.4


### 2.2 Long-Term Care Insurance

After a study of Japan’s long-term care insurance (LTCI) system that began in 2000, a bill related to LTCI for the elderly was passed at the Korean National Assembly plenary session in 2007 which came into effect in July 2008. According to the law, LTCI aims to improve the quality of life of the elderly through physical support and support for daily activities such as housekeeping.

The target population for LTCI is seniors 65 years of age or older, or citizens under the age of 65 but with chronic diseases or disabilities who have had difficulties with daily activities for more than 6 months. LTCI benefits include both in-kind and cash benefits. In-kind benefits include home care and institutional care services. Unlike the National Health Insurance (NHI) program, people can only receive LTCI benefits if they are approved through an eligibility selection process. After the application documents are received, an LTCI representative visits the applicant and evaluates their eligibility through a 90-item LTCI checklist divided into 11 sections. Initially, LTCI beneficiaries were grouped into three categories, with Tier 1 beneficiaries having the most severe disabilities. Now, there are currently 6 levels, with new categories of “special dementia level” added in 2014 and “cognitive support level” added in 2018 (Table 2). In order to be admitted to a nursing home, a beneficiary must be rated at Level 1 or 2. However, with approval by the LTCI committee, even people from levels 3 to 5 may receive an exception to enter a nursing home. The cost of moving into a nursing home is approximately KRW 900,000-1,300,000 (US$800–1,100), and LTCI will cover 80% to 100% of this, taking into account the economic condition of the beneficiary. Furthermore, 85% to 100% of the cost of home services is covered. Long-term care insurance for the elderly is operated as a pay-as-you-go plan, in which business owners, workers, and local subscribers are charged insurance premiums for business expenses as stipulated in the Long-Term Care Insurance Act for the Elderly by business year. Since those who pay for the long-term care insurance premium usually are not eligible for the insurance, the payers effectively subsidize the beneficiaries of the insurance [11,12].

**Table 2:** Eligibility levels for long-term Care.


LEVEL	MENTAL AND PHYSICAL STATUS	LONG-TERM CARE APPROVAL SCORE

1	Requires help in all aspects of daily life	≥95

2	Requires help in most parts of daily life	≥75 and <95

3	Requires help in part of daily life	≥60 and <75

4	Requires some help for daily living because of functional disability	≥51 and <60

5 (Special level of dementia)	Dementia with limited functional decline	≥45 and <51, dementia

6 (Cognition-supporting level)	Dementia with intact physical function	<45, dementia


The total income from long-term care insurance for the elderly increased by an average of 10.7% during the period 2014–18 to KRW 6.3 trillion. At the same time, however, the number of beneficiaries of long-term care benefits reached 670,000 in 2018 with an annual average increase of 12.1% between 2014 and 2018, accounting for 8.8% of the total elderly population. Expenditures for long-term care insurance for the elderly increased by an annual average of 16.1% between 2014 and 2018 to KRW 6.4 trillion, with an increase of 24.2% just in 2018. In addition, the number of patients in nursing facilities supported by the LTCI was 174,534 in 2017, an increase of 12.7% from 2008, and the number of facility users was 159,490, an increase of 14.0% from 2008. The number of home service users was 337,068, an increase of 21.4% compared to 2008.

### 2.3 Finance for Elderly Medical Welfare and Community-oriented integrated care

The Korean government allocates public funds to promote the health of the elderly and to manage their health through the Basic Elderly Care Project and the Elderly Care Comprehensive Project. These projects are being implemented with a focus on areas that cannot be addressed by insurance while playing a complementary role to the above-mentioned insurance. In addition, these projects have a region-oriented operation and are financed by combining the central government and the local government’s funds. An analysis of the Korean central and local government’s budget books as of 2019 shows that the related budget, including both the national and local expenses was KRW 692.2 billion, of which 64.5% was supported by the central government (Table 3). This confirms that a significant amount of financial resources are being supported by local governments. In particular, Table 3 confirms that there are significant regional differences between the proportion of national and local expenditures, the proportion of the elderly population, the proportion of long-term care applications (demand indicator).

**Table 3:** Budget for senior care project by region in Korea (As of 2019, KRW 1 million).


REGION (MAJOR CITY AND PROVINCE)	TOTAL BUDGET	POPULATION SHARE	NATIONAL BUDGET	NATIONAL BUDGET SHARE	MAJOR CITY AND PROVINCE BUDGET	MAJOR CITY AND PROVINCE BUDGET SHARE	COUNTY AND DISTRICT BUDGET	COUNTY AND DISTRICT BUDGET SHARE	SHARE OF THE ELDERLY POPULATION	LONG-TERM CARE INSURANCE APPLICATION SHARE

Gangwon	35,711	5.2	25,121	70.3	2,773	7.8	7,817	21.9	3.8	4.1

Gyeonggi	93,606	13.5	64,462	68.9	3,676	3.9	25,276	27	20.6	19.9

Gyeongnam	65,381	9.4	46,172	70.6	8,149	12.5	11,060	16.9	6.8	7.4

Gyeongbuk	73,504	10.6	54,337	73.9	7,895	10.7	11,272	15.3	6.8	7.7

Gwangju	34,868	5	22,268	63.9	9,978	28.6	2,622	7.5	2.4	3

Dae-gu	36,342	5.3	23,781	65.4	10,378	28.6	2,183	6	4.7	4.3

Daejeon	15,939	2.3	10,752	67.5	4,630	29	557	3.5	2.5	2.8

Busan	62,115	9	37,664	60.6	16,895	27.2	5,706	9.2	7.7	6.3

Seoul	67,398	9.7	16,862	25	42,656	63.3	7,880	11.7	18.4	14.2

Sejong	1,604	0.2	878	54.8	725	45.2	0	0	0.4	0.4

Ulsan	9,402	1.4	6,679	71	1,681	17.9	1,043	11.1	1.6	1.4

Incheon	24,162	3.5	16,065	66.5	4,393	18.2	3,704	15.3	4.8	5

Jeonnam	47,903	6.9	34,113	71.2	4,747	9.9	9,043	18.9	5.3	6.8

Jeonbuk	60,512	8.7	44,072	72.8	7,710	12.7	8,730	14.4	4.6	6.2

Jeju	6,909	1	4,260	61.7	451	6.5	0	0	1.3	1.3

Chungnam	37,874	5.5	26,813	70.8	6,058	16	5,003	13.2	4.8	5.6

Chungbuk	18,997	2.7	12,193	64.2	1,778	9.4	5,026	26.5	3.4	3.6

Total	692,227		446,493	64.5	134,573	19.4	106,921	15.4		


In 2018, South Korean government announced plans to launch the Community Care Projects in communities led by local authorities. One of the main reasons for promoting Community Care is to reorganize the fragmented care services that are expected to run out of LTCI’s finances. The government’s new Community Care initiative provides care that integrates a variety of services. The Ministry of Health and Welfare started providing community care services in June 2019 as a pilot implementation, and plans to expand the service by 2026, when Korea is expected to become a super-aged society, to provide broader coverage to the elderly [12].

Furthermore, the law stipulates that 20% of premium income is to be supported through the national budget for the stability of the operation of state-run health insurance and long-term care insurance. However, in reality, not all of the 20% is supported each year, and instead, 10–19% is supported based on the economic and insurance operation conditions (Table 4).

**Table 4:** Income, Subsidy and Expenditure of NHI and LTCI(KRW 100 million).


		2017	2018	2019	2020

National Health Insurance	Premium income	495,138	533,168	581,010	634,901

	National Subsidy	51,512	54,734	59,721	70,826

	Proportion of National Subsidy	10.4%	10.3%	10.3%	11.2%

	Total Expenditure	571,633	633,199	708,605	770,171

Long-Term Care Insurance	Premium income	32,368	38,525	48,272	63,229

	National Subsidy	5,822	7,107	8,912	12,414

	Proportion of National Subsidy	18.0%	18.4%	18.5%	19.6%

	Total Expenditure	54,839	70,103	82,486	96,759


## 3 Financial Integration for Community Integrated Care

### 3.1 Cases of Financial Integration

Various countries are operating integrated care through financial integration. Service integration through financial integration is showing tangible results. According to an analysis of 38 financial integration systems in 8 countries, 24 out of 38 systems had positive performance in health indicators. 11 schemes did not show any indication of cost reduction, which may be due to the increase in operating cost [13].^2^

In 2014, the United Kingdom established a separate fund called the Better Care Fund to create a foundation for linking medical care and welfare and to develop a new regional model. The Better Care Fund is a program that supports local governments to lay the foundation for linking and integrating health services and social care services with a cross-cutting partnership between the central government entities of NHS England (NHSE) and Department of Health and Social Care (DHSC) and the regional government entities of the Ministry of Housing, Communities and Local Government (MHCLG), and the Local Government Association. The Better Care Fund integrates the central health finance (NHS England: NHSE) social care finance (Improved Better Care Fund: BCF) to provide discretionary funds to local governments for management by local decision-making groups, CCGs (Clinical Commissioning Groups) and local Authorities. In other words, in the case of the United Kingdom, where the health care system is funded by the central government rather than the private insurance system, the establishment of the Better Care Fund provides a mechanism that enables the integration of medical and care finance for the elderly [14].

In Japan, the coverage of long-term care insurance, a type of regional medical insurance, has been expanded to include some medical services within regional comprehensive care. In other words, in the case of Japan, by increasing the contribution of the national and local organizations to regional health insurance and providing social care through the expanded financial resources, the overlap of business is reduced and financial resources are integrated.

With respect to operation, Japan has a governance structure in which the care group takes the lead in making policy proposals and policy-making at the municipal level in meetings run by the existing regional care conferences. A municipality in Japan is considered to have a large population at 50,000 (city) people, which is one-fifth the size of local governments in Korea. Thus, an integrated strategy using governance such as participation in meetings was possible [14].

### 3.2 Directions and Scenarios for Financial Integration for Community Integrated Care in Korea

As in the previous cases, the United Kingdom case provides important implications when considering the direction for Korea’s fiscal consolidation for integrated care. First, the United Kingdom is very similar to Korea in that it provides medical care and social care through public finance. In Korea, medical care and long-term care are provided through the insurance system, but it works like a publicly funded program since insurance premiums are compulsory. In the case of long-term care insurance, premiums are collected from citizens with income, but the benefit is given to the elderly. Therefore, we identify the financial resources available for elderly care by presenting a scenario that integrates the typical United Kingdom financial integration method into the Korean system. In addition, we apply the financial savings factors confirmed through existing research and overseas cases to check the extent and scale of future financial savings.

This study identifies the scale of health insurance, long-term care insurance, and nationally financed elderly care projects, and assumes that the finances are integrated into a new regionally oriented integrated care fund. In the financial integrating care for the elderly, suppose that we shift social inpatients or social admission from health insurance to the local community. As seen in the case of the United Kingdom, if there is an initial or low medical need, the local community reorganizes it in the direction of taking care of it through finance. In the United States, 4.6 million Medicaid subscribers received HCBS as of 2017, and the proportion of Home & Community Based Services (HCBS) expenses increased from 18% to 57% between 1995 and 2016, and the proportion of facility expenses decreased from 82% to 43%. Total healthcare cost savings are estimated to be $41 billion. Even in the case of Program of All-Inclusive Care for the Elderly (PACE-support for those aged 55 and over), which provides a lower level of support than HCBS, which is a strong local community care model, it is effective in reducing medical expense by about 40% compared to facilities [15,16].

Also, in the case of Wako City, Japan, it is reported that the per capita medical expenses decreased from 278,000 yen to 182,000 yen as of 2010 as a result of transferring beneficiaries from nursing hospitals to the local community and carrying out preventive activities. In the case of Korea, it is also reported that the pilot project for specific areas such as Jeonju saved about KRW 1.15 million per person in medical expenses by switching to local care for the target.^3^

We assume that the following resources will be incorporated into the new government fund to fund integrated care before the medical stage. In other words, the new fund is a concept that consolidates the state’s budget and financial resources obtained through insurance (Table 5).

**Table 5:** Finance integration scenario for integrated care in Korea.


SOURCE OF FUND	CATEGORY	(AS OF 2019, KRW 100 MILLION)

		ESTIMATED SIZE OF FUND

		26,860–47,266

Government Subsidy		1,379–4,735

Conversion of insurance income	Level 5 and cognitive level of long-term care insurance	6,837–9,387

	Elective hospitalization group of NHI	4,800–19,300

Transfer of existing Project funded by budget		6,922

	Care Project by Central Government	4,464

	Care Project by Local Government	2,458

1. *Budget for senior care projects operated by the central state and local governments*;
2. *Medical expenses for long-term care insurance for level 5 and cognitive level with the lowest medical needs*;
3. *Medical expenses rather than paid to social inpatients among LTC hospital users covered by health insurance*;
4. *20% of long-term care insurance and health insurance income as stipulated by law (as much as the proportion transferred from long-term care insurance and medical insurance)*.

The logic behind this integration is as follows. First, there are overlapping areas in the business currently run through the national budget and two insurances, which results in the provision of segmented services. Elderly care projects of the national and local governments focus on health care and daily life support in the pre-medical stage. In addition, some subjects of long-term care insurance and health insurance also require management in all stages of medical care, so overlapping areas are occurring.

In particular, as can be seen from research, more than 50% of long-term care insurers are users of the elderly care project, where segmental business integration is possible [17]. In addition, long-term care insurance level 5 and cognitive level subjects have a low desire for medical care or enhanced care, have difficulty entering a nursing facility, and are only provided with home care services. Therefore, this may be integrated into the services provided community care. Since social inpatient group of nursing hospitals is also focused on health management rather than medical treatment, it is possible to design a system to manage it through the integrated care system, and financial integration may be designed for this. Furthermore, we assume that 20% of the premium income used for social inpatients in nursing hospitals and those who are eligible for level 5 and cognitive level of long-term care insurance is provided to the new fund.^4^

If the financial resources for the care projects operated through the national budget in the manner described above and the areas that can be converted to integrated care among the health insurance and long-term care insurance projects are integrated, the size of the fund will be from about KRW 1.7 trillion to KRW 3.5 trillion as of 2019.

### 3.3 Long-term financial outlook through fiscal integration

We now present the amount of financial savings compared to the current system in the long run through the elderly care project through financial integration proposed in the previous section.

First, in the case of the national financial project for caring for the elderly, we combine and confirm the trend of the elderly population and the trend of the elderly welfare budget for 10 years from 2010 (Table 6).^5^

**Table 6:** Fiscal Integration and Fiscal Savings Scenarios.


	NATIONAL BUDGET			LONG TERM CARE		SOCIAL ADMISSION COVERED BY NHI			TOTAL			REDUCTION SCALE		REDUCTION SCALE RATIO	
					
	(1)	(2)	(3)	(4)	(5)	(6)	(7)	(8)	(9)	(10)	(11)	(12)	(13)	(14)	(15)
					
(UNIT: 100 MILLION, %)	EXISTING SCENARIO	MINIMUM ADJUSTMENT SCENARIO	MAXIMUM ADJUSTMENT SCENARIO	EXISTING SCENARIO	ADJUSTMENT SCENARIO	EXISTING SCENARIO	MINIMUM ADJUSTMENT SCENARIO	MAXIMUM ADJUSTMENT SCENARIO	EXISTING SCENARIO	MINIMUM ADJUSTMENT SCENARIO	MAXIMUM ADJUSTMENT SCENARIO	MINIMUM REDUCTION SCALE	MAXIMUM REDUCTION SCALE	MINIMUM REDUCTION RATE	MAXIMUM REDUCTION RATE

2025	9,363	20,325	32,880	159,988	153,183	79,925	72,998	52,073	249,277	246,506	238,136	–2,771	–11,141	–1.1	–4.5

2030	13,510	29,704	47,100	250,491	240,056	110,742	101,144	72,151	374,743	370,904	359,307	–3,839	–15,437	–1.0	–4.1

2035	16,702	40,452	63,908	392,164	376,178	149,317	136,376	97,283	558,183	553,007	537,369	–5,176	–20,814	–0.9	–3.7

2040	19,842	54,659	84,918	617,352	592,552	192,626	175,931	125,499	829,819	823,142	802,969	–6,678	–26,851	–0.8	–3.2

2045	23,841	73,554	111,106	934,271	896,989	239,053	218,335	155,747	1,197,165	1,188,878	1,163,843	–8,287	–33,322	–0.7	–2.8

2050	28,258	95,098	139,867	1,315,697	1,263,677	284,992	260,292	185,678	1,628,947	1,619,067	1,589,221	–9,880	–39,726	–0.6	–2.4


Based on this, the overall budget for welfare for the elderly is expected to reach KRW 67,720 billion in 2050, approximately four times higher than KRW 15,140.8 billion in 2021, taking into account the aging population and the inflation rate. Of this, we conservatively estimate the budget trend of the elderly care project in the future by applying the proportion of KRW 692.2 billion, which is the size of the elderly care project as of 2020. In addition, in the case of long-term care insurance expenditure, we estimate the expenditure for grade 5 and cognitive impairment, taking into account inflation and the proportion of the elderly population. In addition, in the case of health insurance, we apply the proportion of expenditures for nursing hospitals in 2017–2019 based on the long-term outlook for health insurance expenditures of the National Assembly of the Republic of Korea. Through this, when the existing system is applied as it is, the financial projects for the elderly, nursing hospitals, and long-term care expenditures are the same as in column 1, 4 and 6.^6^

Columns 2 and 3 show the scale of health insurance and long-term care insurance when the resources are transferred in the manner described in the previous section. Columns 5 shows the decrease in long-term care insurance spending caused by the transfer of 5th grade and cognitive grade from long-term care insurance to local care services, which is reflected from the transfer of resources used for grade 5 and cognitive grade to the fund. In addition, columns 7 and 8 indicate that the financial resources used for social inpatients in nursing hospitals are transferred to the fund, and insurance expenditures are reduced accordingly. This shows that fiscal integration can save up to 0.6% to 4.5% of the originally projected level. By 2050, the amount of savings is expected to be about KRW 1 trillion to KRW 4 trillion.

These savings come from the following factors: first, the national budget related to the welfare of the elderly is expected to show an average annual increase of 4% compared to the previous increase. However, the national health insurance and long-term care insurance are expected to show an annual average increase of 9% to 7%, respectively. This is believed to be due to the fact that there is competition with other fields in the case of the national budget and there is a mechanism of control through budget deliberation, etc. As a result, there is a structural mechanism whereby the budget does not grow in proportion to demand and service utilization.

However, this forecast is conservative. In other cases, integrated care shows the effect of lowering the utilization of facilities and hospitals [13]. In this study, the effect of reducing and delaying the use of nursing hospitals and facilities through the integration of integrated care projects, namely, financial integration, is not reflected, and additional cost reduction is also possible.

## 4 Conclusion and Policy Implications

This study examines the scale and operation of health insurance, long-term care insurance for the elderly, and national financial projects, which are the three main systems for senior care related projects in Korea, where the population is aging rapidly, and attempts to learn from the experience of the United Kingdom. We predict the scale and the possibility of financial savings when the finances of the project are integrated using methodologies of the existing research. We predict that the prevention and care of the elderly before treatment will require about KRW 1.7 trillion to KRW 3.5 trillion of financial resources as of 2019, under the premise that financial integration will have the same effect. We also confirm that through the integration and operation of these financial resources, it is possible to reduce the financial resources by 0.6% to 4.5%.

This study suggests several policy implications. First, it can be seen that overlapping projects are being carried out for each business entity in social care, which is the stage before medical care and treatment. Although this is a necessary area for both insurance and finance, it can be confirmed that policy effect can be improved through future integration.

Integrated care is being promoted as one of the attempts to overcome these fragmented services. However, empirical analysis and literature analysis have mixed results on whether integrated financing improves cost reduction and service satisfaction [13]. But these results are not a discouragement, but rather clarify the goal to be achieved through careful design. As in other cases of financial consolidation, it is necessary to form a clear cost reduction target and control mechanism and also to have performance indicators to confirm effectiveness. The performance target should be an indicator that can bring health and cost savings for care service recipients, such as reduction in hospitalization rate or delay in hospitalization. There should also be feedback and incentives based on clear evaluation. Since it is quite challenging to analyze its effect, appropriate research and effect analysis design and indicators for it should be established in advance.^4^ In addition, a system that can provide appropriate incentives and additional financial support should be in place in the case of community-oriented financial operation, if care projects tailored to the characteristics of each region are being carried out and there are promising results through this.

Korea is experiencing a high rate of population aging and a low birth rate at the same time and is providing health management, social care, and medical support for the elderly through two insurance systems – health insurance and long-term care insurance – and a substantial national budget. However, the fragmented operation of health insurance and long-term care insurance and the mismatch with the business through the national budget are reducing the effectiveness of the service. In addition, the increase in social hospitalization and the increase in demand will lead to an increase in medical expenses paid through insurance. Therefore, the application of stricter standards for long-term care and health insurance benefits and social hospitalization, along with the adjustment of demand through these standards will reduce medical costs. In addition, if the health care of the elderly is strengthened through community care projects, it can become an important mechanism to delay the use and entry of relatively expensive facilities and hospitals.

Therefore, the source, size, and integration scenario of financial resources used for care for the elderly presented in this study provides an important solution for policy and financial reforms in the field of the elderly care. In addition, we suggest the possibility of integrating disparate financial sources such as budget and insurance based on the type of business and suggesting base line cost reduction based on this integration.

The state and local governments have incentives to implement senior care projects using existing facilities and systems under limited and balanced budgets. However, in the case of long-term care insurance or health insurance, the most striking difference between budget and insurance system is that benefits must be paid when certain conditions are met depending on the nature of the insurance. Accordingly, in the case of the social inpatient group among the long-term care insurance and health insurance, expenditure is expected to increase in proportion to the increase in the elderly population. Therefore, costs are expected be reduced if long-term care insurance and health insurance services are supported through the national budget. Furthermore, the fact that the cost of community-oriented community care is low compared to medical expenses is a factor that enables additional cost reduction.

In order to enjoy the effects suggested by this scenario, some important prerequisites are additionally required in addition to the successful conditions for the general financial consolidation and integrated care operation described above. First, a system that can harmonize primary care, long-term care services, and care services must be established. This will be possible through training and re-education of doctors and social workers, as in the case of Japan. Of course, it is also necessary to analyze various needs and allocate them effectively in key regional centers. In this respect, the existence of infrastructure such as administrative welfare centers in each dong and Public Agency for Social Service established in metropolitan and provincial units is of great significance. In addition, in the expansion and settlement of local community care, it is necessary to establish a mechanism for the care business to reduce medical expenses, and in the process, it is necessary to analyze the mutual effect between the care business and the insurance business. Furthermore, as long-term care insurance coverage is made up of people with high needs for long-term care, it is necessary to further review whether health insurance costs are lowered.

Based on these prerequisites, we confirm that if financial integration for care services and business operation are possible through this, business operation that can reduce the burden of medical expenses with the same effect or in the mid- to long-term is possible.

## Footnotes

https://data.oecd.org/pop/elderly-population.htm.

As a result, the proportion of the central and local governments in the contribution of financial resources for insurance operation has increased. (Central government 25% →39%, prefecture 12.5%→ 19.5%, municipality 12.5%→19.5%, No. 1 premium 22%→22%).

Cases such as the UK show mixed results in reducing costs through community care. However, in that case, it is reported that the increase in administrative costs is the main cause in the operation of the new system. In particular, when elderly people with few medical needs receive care services from the community rather than facilities or hospitals, it appears that the cost reduction is feasible [13].

(Long-term care insurance) Premium income (KRW 4.8 trillion)*Government subsidy ratio (20%)*Ratio of expenditure for grade 5 and cognitive support grade (7%). (Health insurance) Premium income (KRW 58.1 trillion) * State support ratio (20%) * Elective hospitalization group expenditure ratio (0.6%–3.3%).

There is a strong correlation between the elderly welfare budget and the increase in the proportion of the elderly population to the total population. Through regression analysis, we confirm that the budget increases by KRW 116 billion for every 1% point increase in the proportion of the 65-year-old in the population.

The 20% subsidy of the government’s premium income is excluded here because the purpose is to see the size of fiscal savings through fiscal consolidation.
